# Patients with peripheral artery disease demonstrate altered expression of soluble and membrane-bound immune checkpoints

**DOI:** 10.3389/fimmu.2025.1568431

**Published:** 2025-07-21

**Authors:** Rosanne D. Reitsema, Seta Kurt, Ignacio Rangel, Hans Hjelmqvist, Mats Dreifaldt, Allan Sirsjö, Ashok Kumar Kumawat

**Affiliations:** ^1^ Faculty of Medicine and Health, School of Medical Sciences, Örebro University, Örebro, Sweden; ^2^ Department of Clinical Research Laboratory, Faculty of Medicine and Health, Örebro University, Örebro, Sweden; ^3^ Department of Anaesthesia and Intensive Care, Örebro University Hospital, Region Örebro County, Örebro, Sweden; ^4^ Department of Cardiothoracic Surgery, Örebro University Hospital and University Health Care Research Centre, Örebro, Sweden

**Keywords:** peripheral artery disease, intermittent claudication, immune checkpoints, T cells, antigen presenting cells

## Abstract

**Introduction:**

Studies suggest that immune checkpoints play a role in accelerating the formation of atherosclerosis. We aimed to assess the expression of soluble and membrane-bound immune checkpoints in patients with peripheral artery disease (PAD).

**Methods:**

The levels of 14 soluble immune checkpoints were assessed in blood plasma of PAD patients (n= 37) and healthy controls (HCs, n=39) by Multiplex protein assay. The surface expression of immune checkpoints on peripheral blood immune cells was determined by flow cytometry. Cytokine production capacity was measured by flow cytometry in TIM-3+ T cells to determine immune exhaustion.

**Results:**

Soluble levels of PD-L2 were decreased in female PAD patients, whereas soluble levels of TIM-3 showed a trend towards an increased concentration in female PAD patients. PD-L2+ frequencies were higher within all monocyte subsets in PAD patients. CD4+ T cells from PAD patients had increased frequencies of TIM-3+ cells, showing little overlap with other immune exhaustion markers. TIM-3+ CD4+ T cells from both PAD patients and HCs, had a low capacity to produce pro-inflammatory cytokines, but a higher capacity to produce IL-10 compared to TIM-3- CD4+ T cells.

**Conclusion:**

PAD patients show differences in the expression of membrane-bound and soluble immune checkpoints. Some of these differences might be caused by prolonged immune activation, although immune exhaustion markers did not always overlap.

## 1 Introduction

Peripheral artery disease (PAD) is an atherosclerotic disease that is strongly related to ageing. PAD is a significant contributor to cardiovascular morbidity and mortality and is believed to affect more than 230 million people worldwide. PAD affects arteries that perfuse the lower limbs, which leads to claudication and pain, resulting in loss of mobility and quality of life. Loss of lower limbs caused by impaired blood flow is one of the most severe outcomes of PAD (1). PAD is characterized by three different stages, including the asymptomatic stage, the intermittent claudication (IC) stage and the critical limb ischemia (CLI) stage. All stages of PAD, including the asymptomatic stage, are additionally associated with increased cardiovascular disease mortality and morbidity, compared to patients with stable coronary artery disease (CAD) and other forms of cardiovascular disease (CVD) (2).

PAD is caused by atherosclerosis, which is now being recognized as a chronic inflammatory disease affecting the arterial wall (3, 4). The first steps of the inflammatory pathway include endothelial dysfunction and the entering and retention of monocytes in the sub-endothelial space. Transformation of monocytes into macrophages is followed by an immune response against oxidized lipoproteins. Macrophages accumulate lipids intracellularly, which results in the induction of foam cells and more pro-inflammatory cytokine secretion. This leads to the formation of a necrotic core in the vascular wall, which consists of lipids and debris. T cells are involved in the formation of the atherosclerotic plaque as well, as the adaptive immune response against for instance oxidized lipoproteins leads to the aggravation of the pro-inflammatory immune response (5, 6).

Immune checkpoints are key regulators of the immune response. Co-inhibitory immune checkpoint receptor-ligand interactions lead to inhibition of the immune response, whereas stimulatory receptor-ligand interactions activate the immune response. The most well-known immune checkpoint interaction involves the expression of CD28 on T cells and their ligands CD80/CD86 on antigen presenting cells (APCs), which is crucial in activating naïve T cells upon antigen encounter (7). Inhibitory checkpoints such as programmed death-1 (PD-1) are often targeted in immunotherapy to treat cancer. By removing this natural brake on the immune system, T cells become reactivated which is beneficial for anti-tumor immune responses. Not surprisingly, by inhibiting co-inhibitory immune checkpoints, immune-related adverse events can occur related to excessive activation of the T cell response. For instance, cardiovascular events are now being associated with the use of immune checkpoints inhibitors (5, 8). This leads to the hypothesis that proper immune regulation by immune checkpoints is crucial in preventing atherosclerotic diseases.

Immune checkpoints regulate immune responses via their membrane-bound or soluble form. Soluble immune checkpoints are either produced by mRNA expression or are cleaved-of forms of membrane-bound checkpoints and can be measured in plasma. They can interfere with membrane-bound immune checkpoint interactions and either positively or negatively affect these interactions (9). The presence of soluble immune checkpoints therefore forms an additional level of immune regulation. Soluble immune checkpoints can also serve as biomarkers for disease severity. Some checkpoints, such as the co-inhibitory checkpoint and marker for immune exhaustion lymphocyte-activation gene 3 (LAG-3), are associated with clinical characteristics and disease outcomes such as in advanced head and neck cancer (10). In cardiovascular disease, an elevation of the stimulatory checkpoint Glucocorticoid-induced TNFR-related protein (GITR) plasma levels was found compared to healthy controls (HCs) (11). Co-inhibitory immune checkpoints and markers for immune exhaustion have been implicated in atherosclerosis development as well. For instance, T cell immunoglobulin and mucin domain 3 (TIM-3) was previously found to be an important CD8+ T cell regulator in atherosclerosis (12). In addition, the LAG-3 gene was associated with increased risk for myocardial infarction. Lastly, stimulation of the B-and T-lymphocyte attenuator (BTLA) pathway led to reduced lesion development in atherosclerotic mice (13, 14).

The role of immune checkpoints in PAD remains largely undefined, therefore, we aim to assess whether soluble and membrane-bound immune checkpoints are differently expressed in patients with PAD to better understand immune responses in this disease and investigate whether immune checkpoints in PAD are correlated with disease activity.

## 2 Materials and methods

### 2.1 Study population

The study population was part of the PIMM (PAD inflammation, microbiome and metabolites) study at the School of Medical Sciences, Örebro University, Sweden (**Table 1**). Symptomatic PAD patients with intermittent claudication were recruited at the Department of Cardio-thoracic and Vascular Surgery, University Hospital Örebro (USÖ), Sweden, between October 2021 and December 2023. Healthy controls (HCs) that were similar to PAD patients regarding sex and age (+/- 5 years) were recruited at the School of Medical Sciences. Participants with dementia, autoimmune and other inflammatory diseases such as kidney disease, diabetes, irritable bowel syndrome, inflammatory bowel disease, and participants having an active infection, having received antibiotics within two months of study enrolment or any over the counter or prescriptive probiotic or bowel cleansing preparation within the past two months of study enrolment were excluded in this study.

**Table 1:** Clinical characteristics of the study population.

	Soluble immune checkpoints			Membrane-bound immune checkpoints		
	HC (n=39)	PAD (n=37)	p-value	HC (n=27)	PAD (n=24)	p-value
Demographics						
Age, median (years)	72.0	77.0	0.001	72	76	0.057
Sex, M/F	19/20	20/17		14/13	14/10	
Active smoker, n	2	12		1	7	
Previous smoker, n (quit > 1 year)	16	17		11	11	
Non-smoker, n	21	7		15	5	
Clinical parameters (median)						
ABI right	1.21*	0.72	<0.001	1.22*	0.73	<0.001
ABI left	1.20*	0.66*	<0.001	1.21*	0.66*	<0.001
Intima Media Thickness, Max (mm)	0.89†	1.04*	0.001	0.88†	1.04*	0.009
Intima Media Thickness, average (mm)	0.75†	0.85*	0.001	0.73†	0.82*	0.015
Vessel diameter (mm)	5.85†	6.4*	0.006	5.90†	6.20*	0.134
VASCUQOL-6	24†	15†	<0.001	24†	16†	<0.001
Triglycerides (mmol/L)	0.9*	1.2†	0.017	0.9*	1.2†	0.016
Cholesterol (mmol/L)	5.5†	4‡	<0.001	5.25†	4†	<0.001
HDL- Cholesterol (mmol/L)	1.7†	1.3‡	<0.001	1.75†	1.2†	<0.001
LDL- Cholesterol (mmol/L)	3.4†	2.2§	<0.001	3.3†	2.35†	<0.001
Medical history (Y/N)						
Hypertension	7/23	25/0		7/20	24/0	
Hyperlipidemia	4/23	37/0		3/24	24/0	
Previous vascular surgery	0/30	14/10		0/27	13/10	
Heart failure	0/30	3/21		0/27	3/20	
Previous stroke	0/30	5/19		0/27	5/18	
Ischemic heart disease	0/30	7/17		0/27	7/16	
Hypertension medication	5/24	21/2		5/20	20/2	
Antiarrythmics and blood thinning medication	3/27	21/3		3/23	19/3	
Statins	3/27	20/3		2/24	18/3	
Other lipid-lowering drugs	2/28	5/17		2/24	5/16	
Anti-inflammatory treatment/NSAIDs	5/33	5/30		5/21	2/20	
Immunosuppressive therapy	0/38	0/37		0/26	0/24	
	*n=37	*n=36		*n=25	*n=23	
	†n=38	†n=34		†n= 26	†n=22	
		‡n=35				
		§n=33				

M/F, male female; ABI, ankle brachial index; VASCUQOL-6, Vascular Quality of Life Questionnaire-6; Y/N, yes/no.

During recruitment, the participants underwent various non-invasive examinations to assess cardiovascular risk by evaluating microvascular status, vessel diameter and intima media thickness (IMT). Ankle-brachial index (ABI) measurement with pen-Doppler system was performed to assess microvascular status in limbs. IMT of the vessels was evaluated by performing ultrasound of the carotid arteries at the physiology clinic, University Hospital Örebro. To assess the quality of life we have used a PAD-specific health related quality of life questionnaire VascuQoL6. This questionnaire is used in clinical practice for evaluation and in the national quality register SWEDVASC in Sweden.

The present study was carried out in accordance with the principles outlined in the Declaration of Helsinki and was approved by the Swedish Ethical Review Authority (Dnr 2021-03386). Written informed consent was obtained from all the participants.

### 2.2 Preparation of blood samples

From each study participant, whole venous blood was drawn after a fasting period of at least 12 hours. Whole blood samples were either collected in sodium heparin, EDTA or lithium heparin tubes (BD Biosciences, Franklin Lakes, NJ, USA) to perform different analyses. Blood collected in sodium heparin and EDTA tubes were transported immediately at room temperature to our lab and the lithium heparin tubes to the clinical chemistry laboratory at University Hospital Örebro for traditional cardiovascular risk marker analysis.

### 2.3 Soluble immune checkpoints

Soluble immune checkpoints were quantified using the 14 plex ProcartaPlex™ Human Immuno-Oncology Checkpoint Panel 1 (Invitrogen, Waltham, MA, USA) using Bio-Plex^®^ 200 technology (Bio-RAD, Hercules, CA, USA), according to the manufacturer’s instructions. To this end, fasting blood plasma collected in EDTA tubes from 39 HCs and 37 PAD patients was analyzed. This multiplex panel allows simultaneous detection of the co-stimulatory immune checkpoints CD27, CD28, CD80, CD137, GITR and herpesvirus entry mediator (HVEM) and the co-inhibitory checkpoints BTLA, cytotoxic T-lymphocyte associated protein 4 (CTLA-4), indoleamine 2,3- dioxygenase (IDO), LAG-3, PD-1, PD-L1 and PD-L2, and TIM-3. All samples were analyzed in duplicate, and the concentrations of the checkpoint proteins were expressed in pg/mL.

### 2.4 Surface staining membrane-bound immune checkpoints

Next, membrane-bound immune checkpoint expression was measured in sodium heparin blood of 27 HCs and 24 PAD patients by flow cytometry. First, we analyzed the expression of PD-L2 on monocyte subsets (classical: CD14+CD16-, intermediate: CD16dimCD14dim and non-classical: CD14-CD16+) and conventional dendritic cells (cDCs, CD16-CD14-CD11c+). Then, we analyzed the expression of CD28, BTLA, PD-1, TIM-3, LAG-3 and GITR on CD4+ and CD8+ T cells. The distribution of CD4+ and CD8+ differentiation subsets (naïve: CCR7+CD45RA+, central memory: CCR7+CD45RA-, effector memory: CCR7-CD45-, and terminally differentiated: CCR7-CD45RA+) was determined in a subset of patients (n=18) and controls (n=19). Flow cytometry staining was performed in whole blood incubated for 15 minutes at room temperature with monoclonal fluorescent antibodies (**Supplementary Table 1**). Blood was subsequently incubated for 10 minutes with FACS Lysing solution (1X, BD Biosciences). After centrifugation, the samples were washed with PBS + 1% BSA and resuspended in 300 µL PBS + 1% BSA. Samples were analyzed on a Gallios Flow cytometer (Beckman Coulter, Brea, CA, USA). Fluorescence minus one (FMO) controls and unstained controls were used to set the gates (Gating strategy: **Supplementary Figures 1A, B**).

### 2.5 Cytokine production

To determine whether T cells of HCs and PAD patients that expressed markers for immune exhaustion were indeed exhausted, whole sodium heparin blood of 8 HCs and 13 PAD patients was stimulated to measure intracellular cytokine production with flow cytometry. Sodium heparin blood was diluted 1:1 with RPMI 1640 medium (Gibco, Waltham, Massachusetts, US) and stimulated with phorbol 12-myristate 13-acetate (PMA, 50 ng/mL, Sigma‐Aldrich, St. Louis, MO, USA), ionomycin (1,6 µg/mL, Sigma‐Aldrich) and brefeldin A (BFA, 1 µg/mL, BD Biosciences), for four hours at 37˚C + 5% CO2. Control samples were incubated with BFA only. After four hours of stimulation, samples were mixed with Pharm lyse (1X, BD Biosciences) and incubated for 10 minutes at room temperature. Cells were washed once with FACS buffer (PBS + 2% fetal bovine serum (FBS), 2 mM EDTA) and then incubated with diluted fixable viability stain 510 (1:10, BD Biosciences) for 15 minutes. Samples were then stained for surface markers (CD3, CD4, CD8, PD-1 and TIM-3, **Supplementary Figure 1C**, **Supplementary Table 1**) for 15 minutes. Afterwards, samples were washed once with FACS buffer and fixed and permeabilized by adding 500 µL fixation/permeabilization solution (BD Biosciences). After 20 minutes of incubation, samples were centrifuged and then washed and incubated for 10 minutes with Perm/Wash buffer (1X, BD Biosciences). Samples were stained for intracellular cytokines (TNF-α, IL-17A, IL-10 and IFN-γ) for 30 minutes and washed again with Perm/Wash buffer. Samples were resuspended in FACS buffer and analyzed on a Gallios Flow cytometer (Beckman Coulter).

### 2.6 Data analysis

Flow cytometry data were analyzed using Kaluza version 2.1 (Beckman coulter). The levels of soluble immune checkpoints were analyzed using Bio-Plex manager software version 6.2. Results were derived through comparison to a standard curve (5-parameter logistic curve fit) of known concentration of each analyte. Graphs were created and statistical analyses were executed in GraphPad Prism version 10. SPSS version 28 was used for conducting a factorial ANOVA to test for the effect of smoking status on soluble PD-L2 and TIM-3 levels. Statistical significance between HCs and PAD patients was tested using Mann-Whitney U tests. Statistical significance within the groups was determined with Wilcoxon signed-rank tests. For correlation analyses Spearman tests were used. P-values <0.05 were considered statistically significant.

## 3 Results

### 3.1 Patient population characteristics

We included 39 HCs and 37 PAD patients to assess the concentration of soluble immune checkpoints in blood plasma. The PAD patients were older in age than the HCs (p<0.001) and included more active smokers than HCs. The microvascular status was notably impaired in PAD patients, as indicated by a significantly lower median ankle-brachial index (ABI) compared to HCs (**Table 1**). The median vessel diameter of carotid arteries and maximum and average intima media thickness was higher in PAD patients than HCs (**Table 1**). PAD patients had a significantly lower VascuQoL6 score compared to HCs suggesting a declined quality of life in PAD patients (**Table 1**). All PAD patients experienced hypertension and hyperlipidemia, for which most of them are treated with hypertension medication and lipid lowering drugs. Only a small proportion of HCs experienced hypertension and hyperlipidemia. A substantial subgroup of 27 HCs and 24 PAD patients were included in follow-up experiments in which we assessed the expression of membrane-bound immune checkpoints. Within this subgroup, carotid vessel diameter did not significantly differ between groups, whereas other differences in clinical characteristics were similar to those in the larger subset of patients and controls (**Table 1**).

### 3.2 Soluble TIM-3 and PD-L2 concentrations differed between female PAD patients and HCs

**Figure 1:**
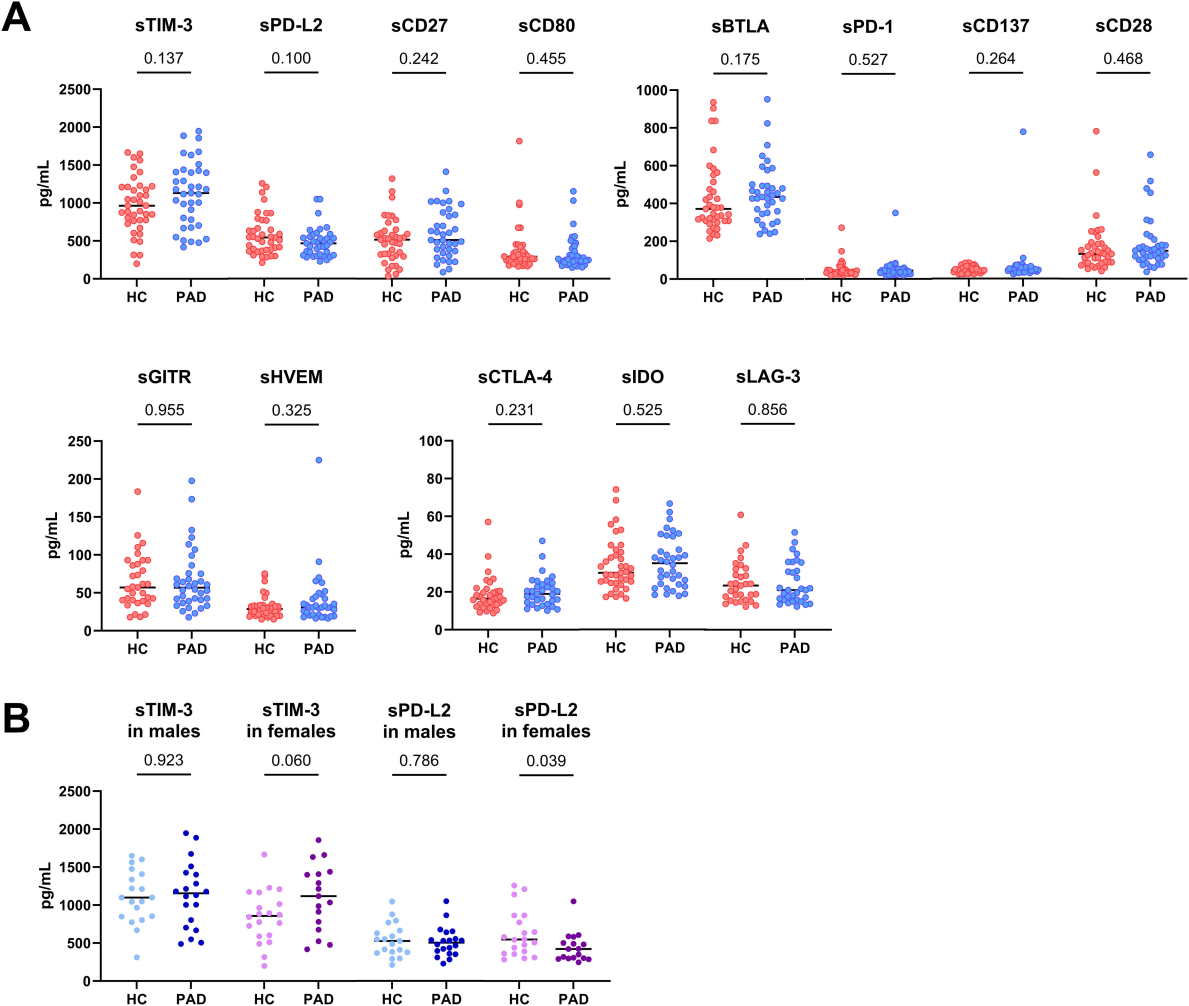
Concentration of soluble immune checkpoints in PAD and HCs. **(A)** The concentration of the measured soluble immune checkpoints was similar between PAD patients (n=37) and HCs (n=39). **(B)** The concentration of soluble TIM-3 was similar between male HCs (n=19) and PAD patients (n=20) but higher in female PAD patients (n=17) than in female HCs (n=20). The concentration of soluble PD-L2 was similar between males but lower in female PAD patients than in female HCs. Horizontal bars reflect the median. Mann-Whitney U tests were used to compare HCs and PAD patients. P-values are shown in the graphs. PAD, peripheral artery disease; HC, healthy controls; TIM-3, T-cell immunoglobulin and mucin domain 3; PD-L2, programmed cell death 1 ligand 2; BTLA, B-and T-lymphocyte attenuator; PD-1, programmed cell death 1; GITR, glucocorticoid-induced TNRF-related protein; HVEM, herpesvirus entry mediator; CTLA-4, cytotoxic T-lymphocyte associated protein 4; IDO, indoleamine 2,3- dioxygenase; LAG-3, lymphocyte-activation gene 3.

We assessed the concentration of soluble immune checkpoints using Bioplex technology in blood plasma (**Figure 1**). The concentration of immune checkpoints was similar between patients and HCs (**Figure 1A**), although large variations could be observed within the groups. The concentration of PD-L1 was too low to be measured in blood plasma and is therefore not shown. Upon stratifying the groups based on sex, we found that female PAD patients had higher concentrations of soluble TIM-3 than female HCs (trend p=0.06), whereas the concentration of soluble TIM-3 was not different between male HCs and PAD patients (**Figure 1B**). Soluble PD-L2 was lower in female PAD patients than HCs and was similar between male HCs and PAD patients (**Figure 1B**).

Since the PAD group was significantly older than the HCs group, we assessed whether age correlated with soluble PD-L2 and TIM-3 concentrations. Additionally, we assessed whether these checkpoints correlated with clinical characteristics. Age did not correlate with either soluble TIM-3 or PD-L2 concentrations in PAD patients. Smoking status had no effect on soluble TIM-3 or PD-L2 concentrations either (p=0.320 and p=0.608, respectively). Notably, soluble PD-L2 levels were positively correlated with average intima media thickness (R=0.34, p=0.046). Soluble TIM-3 concentrations did not correlate with clinical characteristics.

### 3.3 Increased proportions of membrane-bound PD-L2 on monocytes, TIM-3 on CD4+ and LAG-3 on CD8+ T cells in PAD patients

Since soluble PD-L2 and TIM-3 concentrations were different between female PAD patients and HCs, we assessed the expression of their membrane-bound forms in APCs and T cells. We additionally assessed the expression of PD-1 on T cells. The expression of GITR, TIM-3, LAG-3 and BTLA on T cells was also examined, as these checkpoints have previously been implicated in atherosclerosis, as mentioned earlier. CD28 was included because its absence typically indicates immune aging or senescence.

**Figure 2:**
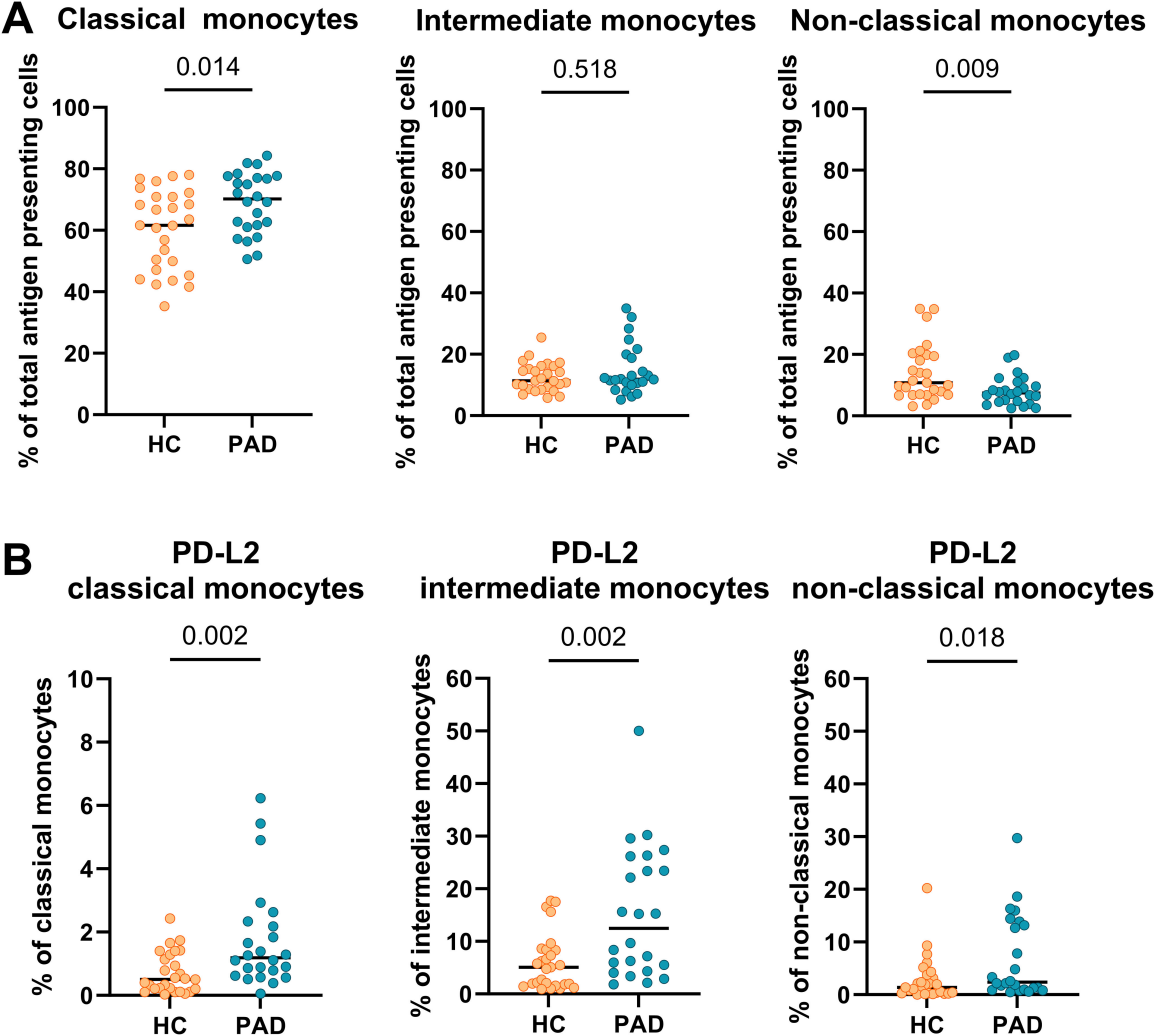
Frequencies of monocytes and expression of PD-L2 within monocyte subsets. **(A)** The frequency of classical monocytes was higher, and the frequency of non-classical monocytes was lower in PAD patients (n=24) than in HCs (n=27). **(B)** PD-L2 frequencies were higher within all monocyte subsets in PAD patients than in HCs. Horizontal bars reflect the median. Mann-Whitney U tests were used to compare HCs and PAD patients. P-values are shown in the graphs. PAD, peripheral artery disease; HC, healthy controls; PD-L2, programmed cell death 1 ligand 2.

The distribution of monocyte-subsets was different between PAD patients and HCs (**Figure 2A**). The frequencies of classical monocytes were higher in PAD patients than in HCs. Intermediate monocyte-frequencies were similar between the groups, and the frequency of non-classical monocytes was lower in PAD patients. Within each monocyte subset, the frequencies of cells expressing PD-L2 were higher in PAD patients than in HCs (**Figure 2B**). This pattern was similar in males and females between HCs and PAD patients, but the analyses lacked statistical power due to the smaller group sizes (**Supplementary Figure 2**). PD-L2+ frequencies within conventional DCs were similar between PAD and HCs (**Supplementary Figure 3**).

**Figure 3:**
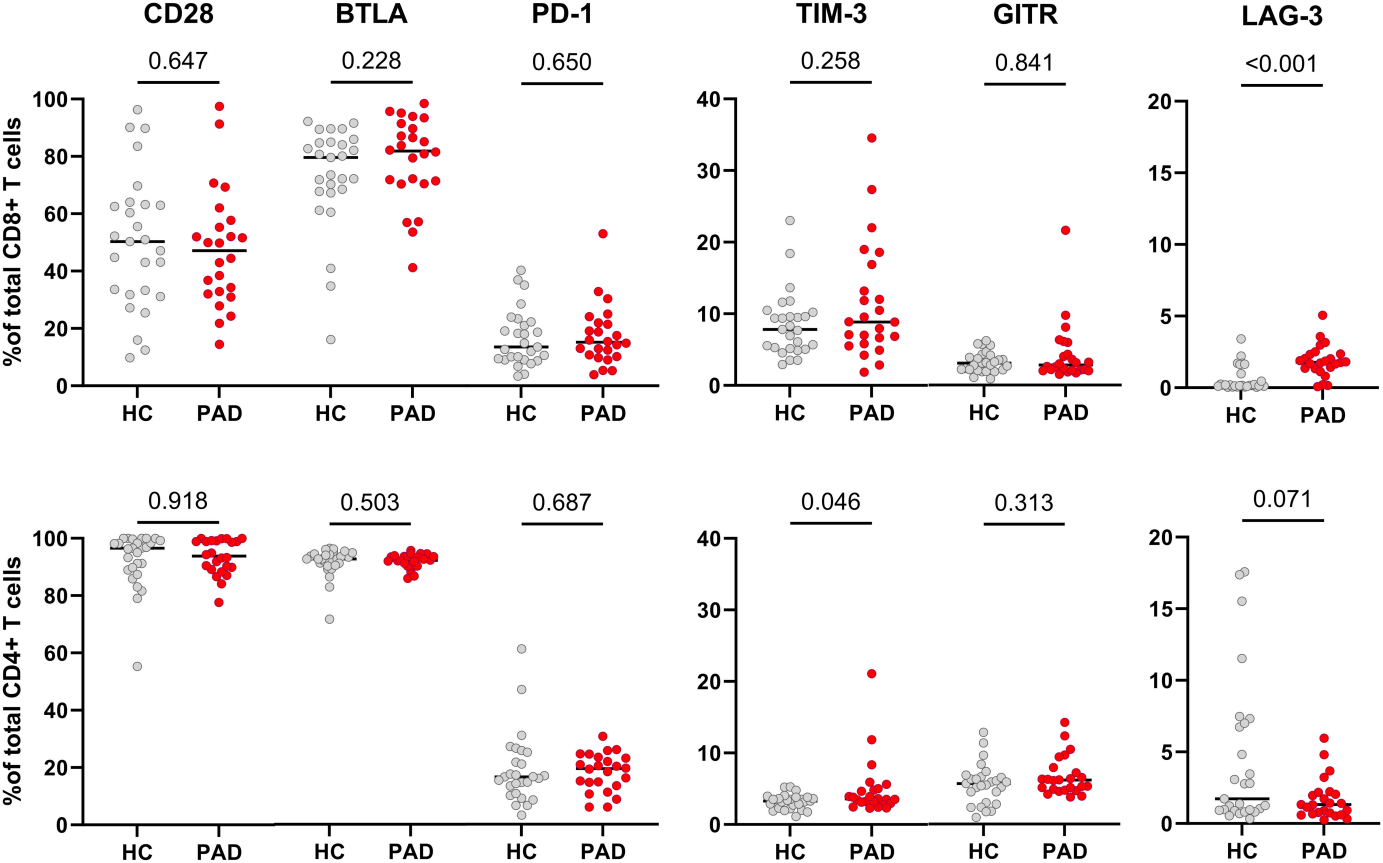
Expression of membrane-bound immune checkpoint receptors in CD8+ and CD4+ T cells of HCs and PAD patients. The upper panel shows the frequencies of cells expressing CD28, BTLA, PD-1, TIM-3, GITR and LAG-3 within CD8+ T cells of HCs (n=27) and PAD patients (n=24). The lower panel shows the frequencies of these checkpoints within CD4+ T cells. Horizontal bars reflect the median. Mann-Whitney U tests were used to compare HCs and PAD patients. P-values are shown in the graphs. PAD, peripheral artery disease; HC, healthy controls; BTLA, B-and T-lymphocyte attenuator; PD-1, programmed cell death 1; TIM-3, T-cell immunoglobulin and mucin domain 3; GITR, glucocorticoid-induced TNRF-related protein; LAG-3, lymphocyte-activation gene 3.

Next, we assessed the expression of immune checkpoint receptors on CD8+ and CD4+ T cells. We found the frequencies of cells expressing CD28, BTLA, PD-1 and GITR to be similar within CD8+ and CD4+ T cells of HCs and PAD patients (**Figure 3**). The frequency of TIM-3-expressing cells within CD8+ T cells was similar between HCs and PAD patients but higher in CD4+ T cells of PAD patients (**Figure 3**). Although the difference in soluble TIM-3 concentration was mainly observed in female HCs and PAD patients, we found no similar trend in membrane-bound TIM-3 expression. Finally, LAG-3 was virtually absent on CD8+ T cells of HCs, but elevated in PAD patients (**Figure 3**). Importantly, LAG-3 negatively correlated with vessel diameter in PAD patients (R=-0.41, P=0,05). Since the expression of immune checkpoints can differ across CD4+ and CD8+ T cell differentiation subsets, we analyzed the distribution of these subsets in a smaller, randomly selected subset of patients and controls. We observed no differences in the distribution of either CD4+ or CD8+ differentiation subsets between the groups (**Supplementary Table 2**).

### 3.4 Co-expression analyses of immune checkpoints reveal differences between HCs and PAD patients

**Figure 4:**
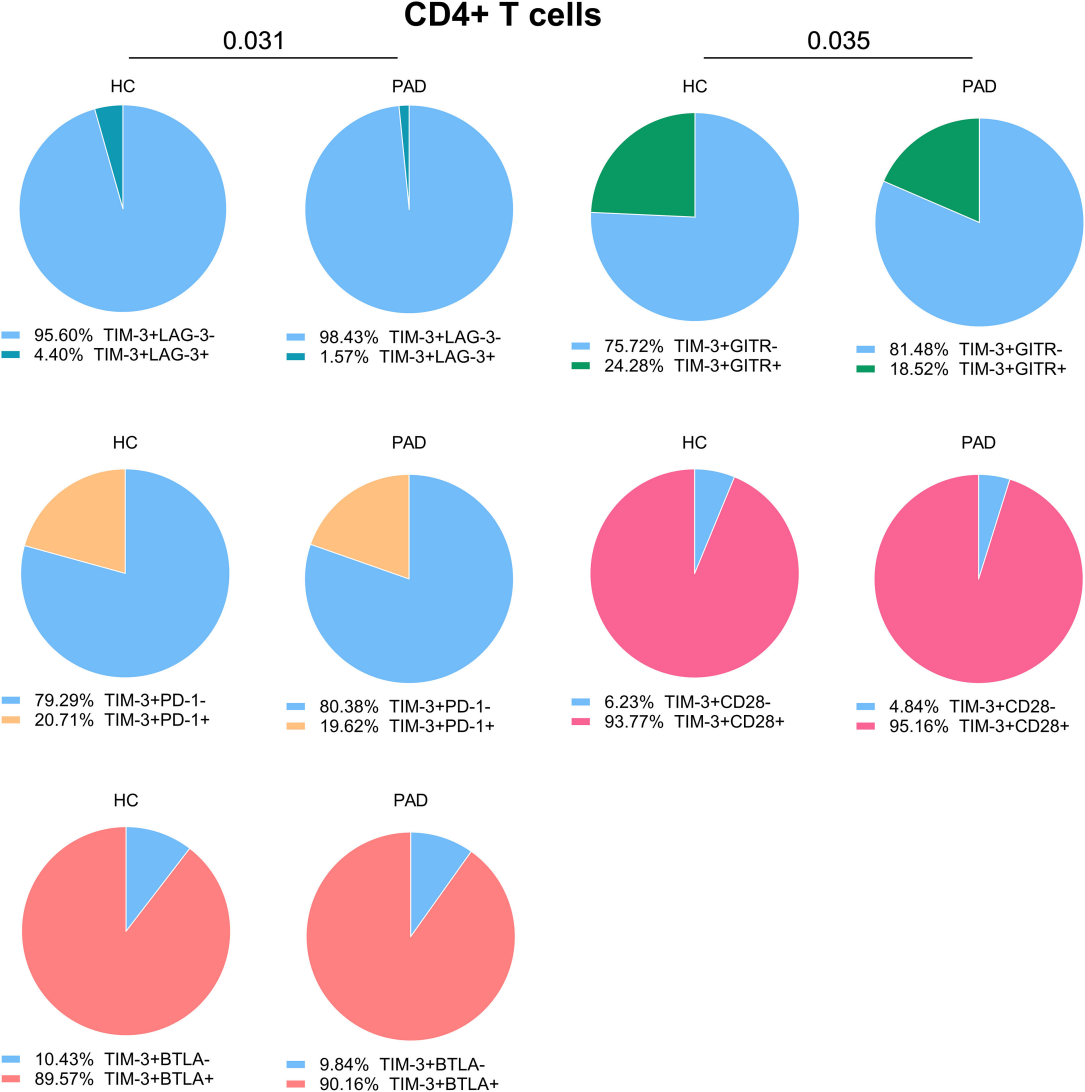
Co-expression of TIM-3 with other immune checkpoints in CD4+ T cells. We show the relative co-expression of TIM-3+ cells with other immune checkpoints within CD4+ T cells in PAD patients (n=24) and HCs (n=27). Shown are the frequencies of total TIM-3+ cells that do or do not express the other indicated immune checkpoints. Mann-Whitney U tests were used to compare HCs and PAD patients. P-values are shown in the graphs. PAD, peripheral artery disease; HC, healthy controls; BTLA, B-and T-lymphocyte attenuator; PD-1, programmed cell death 1; TIM-3, T-cell immunoglobulin and mucin domain 3; GITR, glucocorticoid-induced TNRF-related protein; LAG-3, lymphocyte-activation gene 3.

As the frequency of TIM-3+ cells within CD4+ T cells was higher in PAD patients than in HCs, we assessed whether co-expression with other checkpoints was different between the groups as well. We found that the majority of TIM-3+ CD4+ T cells were negative for LAG-3, and this was even more pronounced in PAD patients. The relative frequency of TIM3+GITR+ was lower in PAD patients than in HCs. Around 20% of TIM-3+ cells expressed PD-1 as well, and almost all TIM-3+ cells were also positive for CD28 and BTLA (**Figure 4**).

Previous analyses show that CD8+ T cells have a higher frequency of LAG-3 expressing cells in PAD patients than in HCs. Most LAG-3+CD8+ T cells were TIM-3 negative, although the relative percentage of LAG-3+TIM-3+ CD8+ T cells was slightly higher in PAD patients. LAG-3+ CD8+ T cells were largely negative for GITR and PD-1 expression as well. About half of the LAG-3+ CD8+ T cells co-expressed CD28 and nearly all expressed BTLA (**Supplementary Figure 4**). These results indicate that exhaustion or inhibition markers such as PD-1, TIM-3, BTLA and LAG-3 are not all co-expressed on the same cells.

### 3.5 TIM-3+ CD4+ T cells are exhausted in PAD patients and HCs

**Figure 5:**
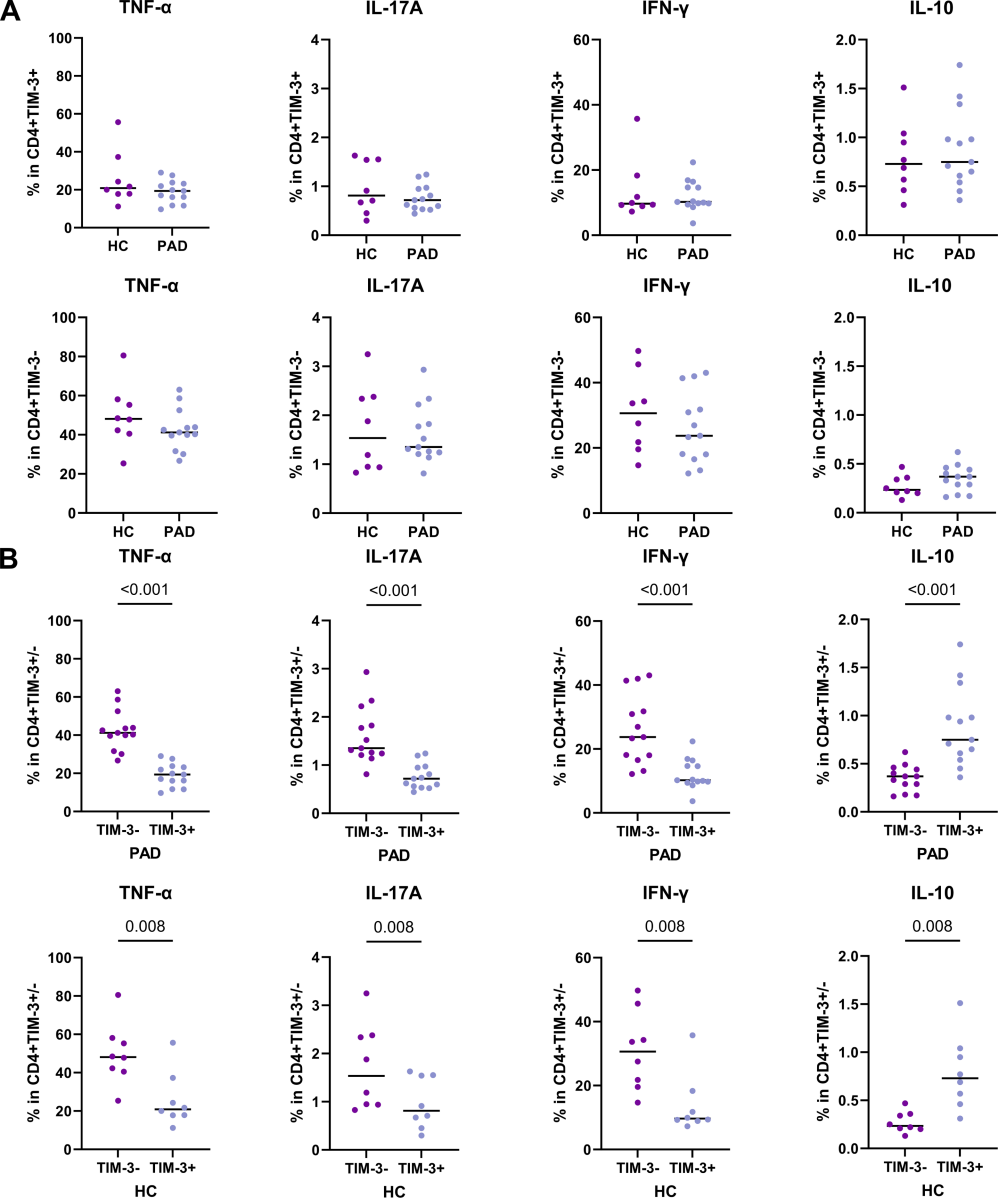
Cytokine production by TIM-3+ CD4+ T cells. Intracellular cytokine production after stimulation of CD4+ T cells was assessed in PAD patients (n=13) and HCs (n=8). **(A)** The total frequency of TIM-3+CD4+ T cells expressing intracellular TNF-α, IFN-γ, IL-17A and IL-10 are shown in the upper panel. The lower panel shows the frequency of TIM-3-CD4+ T cells expressing intracellular TNF-α, IFN-γ, IL-17A and IL-10. **(B)** The pair-wise comparison of intracellular TNF-α, IFN-γ, IL-17A and IL-10-expressing cells within TIM-3- and TIM-3+ CD4+ T cells is shown within the PAD patients and HCs. Horizontal bars reflect the median. Mann-Whitney U tests were used to compare HCs and PAD patients. Wilcoxon signed-rank tests were used to compare within the groups. P-values are shown in the graphs. PAD, peripheral artery disease; HC, healthy controls; TIM-3, T-cell immunoglobulin and mucin domain 3; TNF-α, Tumor necrosis factor alpha; IFN-γ, Interferon gamma.

Our results show that the frequency of TIM-3+CD4+ T cells was higher in PAD patients. A small subset of these cells co-expressed exhaustion markers such as LAG-3 and PD-1, while the majority expressed BTLA and CD28. We therefore analyzed whether TIM-3+ cells are exhausted, as defined by the reduced capacity to produce (pro)-inflammatory cytokines. We show that TIM-3+CD4+ T cells of PAD patients and HCs had similar cytokine production capacity (**Figure 5A**). Compared to TIM-3- CD4+ T cells, TIM-3+CD4+ T cells of both PAD patients and HCs had lower frequencies of cells expressing TNF-α, IFN-γ and IL-17A. However, they had a relatively higher frequency of IL-10+ cells (**Figure 5B**). These findings indicate that a significant subset of TIM-3+ CD4+ T cells indeed displays characteristics consistent with exhaustion.

## 4 Discussion

In this study, we aimed to investigate whether concentrations of soluble immune checkpoints and the expression of membrane-bound immune checkpoints were different between PAD patients and HCs. We found that soluble immune checkpoints and membrane-bound immune checkpoints show different expression patterns between PAD patients and HCs.

At first glance, soluble immune checkpoint concentrations appear to be largely similar between PAD patients and HCs. However, after further analysis we show that there is a sex-difference in soluble immune checkpoint concentrations between PAD patients and HCs, as soluble PD-L2 was lower in female PAD patients and soluble TIM-3 showed a trend to be higher in female PAD patients than in female HCs. The effect of sex on immune checkpoint expression was previously assessed in a study on young and older healthy donors. In that study, sex especially affected the expression of PD-1 expression (15). We are not aware of studies showing differences between females and males regarding soluble immune checkpoint concentrations. Although soluble levels of PD-L2 were lower in female PAD patients, soluble PD-L2 positively correlated with intima media thickness. Intima media thickness is a marker for disease severity, but since the levels of PD-L2 were lower in female PAD patients, it is challenging to interpret this correlation.

Next, we show that the frequency of membrane-bound PD-L2-expressing cells was higher in monocyte subsets of PAD patients. Membrane-bound PD-L2 expression frequencies were not different between female and male PAD patients and controls. These results align with previous findings in systemic lupus erythematosus (SLE), where the authors showed that PD-L2 expression on monocytes was higher and soluble PD-L2 levels were lower in SLE patients than in HCs (16). Although soluble PD-L2 is most likely controlled by post-transcriptional regulation through alternative splicing (17), further research is necessary to better understand the specific cellular sources and post-transcriptional processing mechanisms. Such insights are crucial for interpreting the observed discrepancy between increased surface expression and reduced plasma levels of PD-L2 in PAD patients.

The role of the PD-1-PD-L1/PD-L2 axis in atherosclerosis is still up to debate. A previous study showed that the PD-1-PD-L1/PD-L2 axis was protective in preventing atherosclerotic lesion formation in mice (18). However, another study in human adult cancer patients with atherosclerotic plaques showed that targeting PD-1 reduces atherosclerotic plaque size. Plaque-specific PD-1+ T cells appeared to be pro-inflammatory instead of exhausted. These cells also did not express other markers of exhaustion, such as LAG-3 and TIM-3. The authors stated that this pro-inflammatory state could be caused by the lack of PD-L1 and PD-L2 expression in the atherosclerotic plaque. Without being able to bind to PD-1 ligands, PD-1+ T cells will not receive inhibitory signals that will lead to immune suppression. The monoclonal antibody acted as a substitute PD-1 ligand, which suppressed the pro-inflammatory functions of PD-1+ T cells, thereby reducing plaque size (19). This study emphasizes that it would be important to investigate PD-1 and PD-L1/PD-L2 expression in plaques from PAD patients as well, to assess whether the local tissue expression of these immune checkpoints differ from our findings in blood.

Further inspection of immune checkpoint expression on T cells revealed that the frequency of TIM-3+ was higher in CD4+ T cells whereas the frequency of LAG-3+ cells was higher in CD8+ T cells of PAD patients. LAG-3 has previously been targeted in an atherosclerotic mouse model. Here, plaque size was not affected in LAG-3 knockout mice or by blocking LAG-3 with monoclonal antibodies had no effects on plaque size. However, the levels of pro-inflammatory CD4+ T cells increased and accumulated more in plaques of mice lacking LAG-3 (20). LAG-3 could therefore be an important regulator of T cell activation in atherosclerosis. In our study, the increased expression could indicate a regulatory mechanism in PAD patients to counteract possible increased CD8+ T cell responses. This is also emphasized by the fact that LAG-3 correlated negatively with vessel diameter in PAD patients.

TIM-3 expression was increased in CD4+ T cells of PAD patients. In a mouse model of atherosclerosis, blocking TIM-3 reduced lesion size and increased monocyte and CD4+ T cell frequencies (21). In PAD patients with intermittent claudication, only an increase in PD-1+TIM-3+CD8+ T cells was reported (12). This study was performed in patients before start of treatment, which could potentially explain the differences in outcome with our study. Another study showed that expression of TIM-3+CD4+ T cells was increased in coronary heart disease patients as well and correlated with disease severity (22). In our study, the expression of TIM-3 did not correlate with clinical characteristics of PAD patients. We also show that a large percentage of TIM-3+CD4+ T cells lacked expression of other exhaustion markers, both in PAD patients as in HCs. However, a large percentage of TIM-3+ cells showed signs of exhaustion. The increased expression in PAD could therefore be a consequence of prolonged immune activation.

The main limitation of our study is that we included PAD patients that are currently being treated for hypertension and hyperlipidemia. Therefore, we could not determine whether immune checkpoint expression was associated with markers of hypertension or lipid levels. Additionally, the modest sample size may have limited our statistical power, particularly when stratifying participants by sex. Another limitation is the absence of plaque-specific immune cell data. Future studies should aim to assess immune checkpoint expression within atherosclerotic plaques, using techniques such as immunofluorescence or immune cell isolation from plaque tissue, to gain deeper insights into the local immune landscape and pathogenesis of PAD.

In conclusion, we show that PAD patients exhibit altered expression of both membrane-bound and soluble immune checkpoints, particularly of those associated with immune exhaustion. Although exhaustion markers did not always overlap, their overall increase may reflect chronic immune activation in PAD. Differences in soluble PD-L2 and TIM-3 were only found between female HCs and PAD patients, emphasizing the importance of the effects of sex on immune checkpoint expression. Although we found no clear associations between immune checkpoint expression and clinical disease activity, the observed increase in LAG-3, correlating negatively with vessel diameter, may indicate a compensatory regulatory mechanism aimed at limiting inflammation. Future studies should be focused on investigating immune checkpoint expression in atherosclerotic lesions of PAD patients and explore whether individuals with advanced disease, such as critical limb ischemia, exhibit more pronounced signs of immune exhaustion.

## Data Availability

The raw data supporting the conclusions of this article will be made available by the authors, without undue reservation.

## References

[1] Aday AW Matsushita K. Epidemiology of peripheral artery disease and polyvascular disease. Circ Res. (2021) 128:1818–32. doi: 10.1161/CIRCRESAHA.121.318535, PMID: 34110907 PMC8202714

[2] Sigvant B Lundin F Wahlberg E. The risk of disease progression in peripheral arterial disease is higher than expected: A meta-analysis of mortality and disease progression in peripheral arterial disease. Eur J Vasc Endovascular Surgery. (2016) 51:395–403. doi: 10.1016/j.ejvs.2015.10.022, PMID: 26777541

[3] Hansson GK. Inflammation, atherosclerosis, and coronary artery disease. New Engl J Med. (2005) 352:1685–95. doi: 10.1056/nejmra043430, PMID: 15843671

[4] Hansson GK Libby P. The immune response in atherosclerosis: A double-edged sword. Nat Rev Immunol. (2006) 6:508–19. doi: 10.1038/nri1882, PMID: 16778830

[5] Piras L Zuccanti M Russo P Riccio F Agresti A Lustri C. Association between immune checkpoint inhibitors and atherosclerotic cardiovascular disease risk: another brick in the wall. Int J Mol Sci. (2024) 25(5):2502. doi: 10.3390/ijms25052502, PMID: 38473748 PMC10931678

[6] Tabas I Lichtman AH. Monocyte-macrophages and T cells in atherosclerosis. Immunity. (2017) 47:621–34. doi: 10.1016/j.immuni.2017.09.008, PMID: 29045897 PMC5747297

[7] Chen L Flies DB. Molecular mechanisms of T cell co-stimulation and co-inhibition. Nat Rev Immunol. (2013) 13:227–42. doi: 10.1038/nri3405, PMID: 23470321 PMC3786574

[8] Laenens D Yu Y Santens B Jacobs J Beuselinck B Bechter O. Incidence of cardiovascular events in patients treated with immune checkpoint inhibitors. J Clin Oncol. (2022) 40:3430–8. doi: 10.1200/JCO.21.01808, PMID: 35772044

[9] Gu D Ao X Yang Y Chen Z Xu X. Soluble immune checkpoints in cancer: Production, function and biological significance. J ImmunoTherapy Cancer. (2018) 6:132. doi: 10.1186/s40425-018-0449-0, PMID: 30482248 PMC6260693

[10] Botticelli A Zizzari IG Scagnoli S Pomati G Strigari L Cirillo A. The role of soluble lag3 and soluble immune checkpoints profile in advanced head and neck cancer: A pilot study. J Pers Med. (2021) 11(7):651. doi: 10.3390/jpm11070651, PMID: 34357118 PMC8304359

[11] Bosmans LA Shami A Atzler D Weber C Gonçalves I Lutgens E. Glucocorticoid induced TNF receptor family-related protein (GITR) – A novel driver of atherosclerosis. Vasc Pharmacol. (2021) 139:106884. doi: 10.1016/j.vph.2021.106884, PMID: 34102305

[12] Qiu MK Wang SC Dai YX Wang SQ Ou JM Quan ZW. PD-1 and Tim-3 pathways regulate CD8+ T cells function in atherosclerosis. PloS One. (2015) 10(6):e0128523. doi: 10.1371/journal.pone.0128523, PMID: 26035207 PMC4452700

[13] Rodriguez A. High HDL-cholesterol paradox: SCARB1-LAG3-HDL axis. Curr Atheroscl Rep. (2021) 23:5. doi: 10.1007/s11883-020-00902-3, PMID: 33398433 PMC7782461

[14] Douna H Amersfoort J Schaftenaar FH Kröner MJ Kiss MG Slütter B. B- And T-lymphocyte attenuator stimulation protects against atherosclerosis by regulating follicular B cells. Cardiovasc Res. (2020) 116:295–305. doi: 10.1093/cvr/cvz129, PMID: 31150053

[15] Reitsema RD Hid Cadena R Nijhof SH Abdulahad WH Huitema MG Paap D. Effect of age and sex on immune checkpoint expression and kinetics in human T cells. Immun Ageing. (2020) 17:32. doi: 10.1186/s12979-020-00203-y, PMID: 33292359 PMC7640492

[16] Tong M Fang X Yang J Wu P Guo Y Sun J. Abnormal membrane-bound and soluble programmed death ligand 2 (PD-L2) expression in systemic lupus erythematosus is associated with disease activity. Immunol Lett. (2020) 227:96–101. doi: 10.1016/j.imlet.2020.09.001, PMID: 32891685

[17] He XH Liu Y Xu LH Zeng YY. Cloning and identification of two novel splice variants of human PD-L2. Acta Biochim Biophys Sin (Shanghai). (2004) 36:284–9. doi: 10.1093/abbs/36.4.284, PMID: 15253154

[18] Bu DX Tarrio M Maganto-Garcia E Stavrakis G Tajima G Lederer J. Impairment of the programmed cell death-1 pathway increases atherosclerotic lesion development and inflammation. Arterioscler Thromb Vasc Biol. (2011) 31:1100–7. doi: 10.1161/ATVBAHA.111.224709, PMID: 21393583 PMC3104026

[19] Fan L Liu J Hu W Chen Z Lan J Zhang T. Targeting pro-inflammatory T cells as a novel therapeutic approach to potentially resolve atherosclerosis in humans. Cell Res. (2024) 34:407–27. doi: 10.1038/s41422-024-00945-0, PMID: 38491170 PMC11143203

[20] Mulholland M Kritikou E Katra P Nilsson J Björkbacka H Lichtman AH. LAG3 regulates T cell activation and plaque infiltration in atherosclerotic mice. JACC CardioOncol. (2022) 4:635–45. doi: 10.1016/j.jaccao.2022.09.005, PMID: 36636446 PMC9830219

[21] Foks AC Ran IA Wasserman L Frodermann V Ter Borg MND De Jager SCA. T-cell immunoglobulin and mucin domain 3 acts as a negative regulator of atherosclerosis. Arterioscler Thromb Vasc Biol. (2013) 33:2558–65. doi: 10.1161/ATVBAHA.113.301879, PMID: 23990206

[22] Zhang J Zhan F Liu H. Expression level and significance of tim-3 in CD4+ T lymphocytes in peripheral blood of patients with coronary heart disease. Braz J Cardiovasc Surg. (2022) 37:350–5. doi: 10.21470/1678-9741-2020-0509, PMID: 34236813 PMC9162406

